# A modified homogeneity index for multi- target radiotherapy plans by subtracting high-dose subvolumes

**DOI:** 10.7717/peerj.21005

**Published:** 2026-03-20

**Authors:** Yong Sang, Jun Dang, Jianan Wu, Jiajun Cai, Xiaoye Su, Yanling Wu

**Affiliations:** Cancer Hospital & Shenzhen Hospital, Chinese Academy of Medical Sciences and Peking Union Medical College, Shenzhen, Guangdong, China

**Keywords:** Radiotherapy planning, Generalized homogeneity index, Evaluation, Multi-target plans

## Abstract

**Background and Purpose:**

Considering the limitations of current homogeneity index (HI) in multi-target plans, we have redefined the volume (V)_PTV_ in the formula for calculating the HI value of target volumes. This redefinition could provide a reference for clinical applications.

**Methods and Material:**

Since the three widely used HI formulas may not fully capture dose homogeneity in multi-target treatment plans, we have redefined the V_PTV_ in the calculation formulas, which may allow for a better assessment of HI for the multi-target radiotherapy plans. Fifteen clinically treated breast cancer (BC) with conserving surgery patients and fifteen nasopharyngeal carcinoma (NPC) patients were chosen. We calculated the V_PTV_ with the name of V_PTVpro_ and the HI_pro1_, HI_pro2_, HI_pro3_. A paired, two-tailed non-parametric Wilcoxon signed-rank test was utilized to compare V_PTVori_ and V_PTVpro_, HI_ori1_ and HI_pro1_, HI_ori2_ and HI_pro2_, HI_ori3_ and HI_pro3_. The correlations between HI_ori1_ and HI_pro1_, HI_ori2_ and HI_pro2_, HI_ori3_ and HI_pro3_ were also analyzed. We conducted a questionnaire survey among a panel of six senior radiation oncologists and physicists to validate the clinical applicability of this study.

**Results:**

For Planning Target Volume (PTV) of BC and PTV1 of NPC, the V_PTVpro_ were significantly smaller than the V_PTVori_ and HI_pro_ were significantly lower than HI_ori_. The correlation coefficient values of HI_ori1_-HI_pro1_, HI_ori2_-HI_pro2_, and HI_ori3_-HI_pro3_ showed variations, though all demonstrated positive correlations with statistically significant p-values <0.01. The results for these two target volumes revealed a consistent pattern: the HI_ori1_-HI_pro1_ pair exhibited the highest correlation coefficient values, while HI_ori2_-HI_pro2_ consistently showed the lowest values. Meanwhile, clinical validation by the expert panel found that the V_PTVpro_ and HI_pro_ in this study do not affect clinical plan selection. All experts agreed that, compared to V_PTVori_ and HI_ori_, V_PTVpro_ and HI_pro_ better align with their clinical practice for evaluating multi-target radiotherapy plans.

**Conclusions:**

The V_PTVpro_ calculation subtracts the overlapping high-dose target volume from the low-dose target volume. This adjustment is intended to enable a more accurate assessment of dose HI specifically within the low-dose target. Additionally, the V_PTVpro_ is also applicable for HI calculation in single-target volume. We suggest using the V_PTVpro_ to calculate the HI of the target for multi-target plans such as BC with conserving surgery and NPC. Meanwhile, we recommend employing formula 1 for the calculation of HI among the three formulas.

## Introduction

The goal of radiotherapy is to deliver the prescribed dose to the target volume while keeping the dose to surrounding organs at risk (OARs) and normal tissues as low as possible. With advances in medical imaging technology and computational software, multiple radiotherapy plans can now be rapidly generated for the same patient. However, analyzing each treatment-related parameter is complex and time-consuming, making it challenging for radiation oncologists to select the optimal treatment plan. Therefore, there is a critical need to develop efficient tools that can integrate and analyze these data comprehensively. The Homogeneity Index (HI) serves as an ideal tool in this context. As a rapid and straightforward scoring metric, it can effectively analyze and quantify dose uniformity within the target volume, providing valuable reference criteria for clinical plan selection. Therefore, the HI can be utilized to evaluate and compare dose distributions across different treatment plans, facilitating the selection of the optimal plan among available options. Additionally, it serves as a valuable tool for comparing different radiotherapy devices and techniques, providing guidance for the development of future technologies and treatment protocols. This contributes to identifying potential improvements in treatment planning strategies.

During the development of HI, many scholars have proposed different formulas to calculate HI values (Patel et al., 2020). Currently, there are three widely used HI formulas in clinical practice: the first HI formula was proposed by Wu et al. (2003), the second HI formula was introduced by Wang et al. (2005) and the third HI formula was recommended in ICRU Report 83 (International Commission on Radiation Units and Measurements (ICRU), 2010), which shares similarities with the formula proposed by Wu et al. (2003). The treatment plan for patients undergoing radiotherapy can have a variable number of target volumes. Some plans involve a single target volume, such as those for rectal cancer, while others involve multiple target volumes, such as breast cancer (BC). For plans with multiple target volumes, the spatial relationship between each target volume and the prescribed doses can be complex. It may be a complete inclusion relationship, an independent relationship, a small overlap relationship or a mixed relationship that includes both complete inclusion and partial inclusion, as well as independentce. For multi-target plans, the prescription dose for each target may also be different. The three HI formulas can obtain good results on the homogeneity of the Planning Target Volume (PTV) when calculating single target or the multi target plans that had a small overlap relationship with the same prescription such as PTVsc and PTVcw of BC. However, when evaluating multi-target, multi-prescription plans, such as PTV1 of NPC, the dose metrics D_2%_, D_5%_, D_50%_, D_95%_ and D_98%_ used in these formulas are influenced by the combined dose distribution of PTVp, PTVn, and PTVrpn. Consequently, the calculated HI may be distorted due to this cross-target dose interference. The calculated HI value may be also distorted and cannot represent the true homogeneity of the target PTV1 in the plan. Therefore, we have redefined volume (V)_P__TV_ in the published HI formula, so that its HI value may more accurately reflect the target homogeneity of multi-target planning. And we demonstrated its application in the volumetric modulate arc therapy (VMAT) plan for BC patients undergoing breast-conserving surgery and NPC patients, and studied its relationship with the published three HI calculation formulas.

## Terminology and definitions

V_PTVori_: The original planning target volume, referring to the clinically defined target volume obtained through direct contouring or expansion from Gross Tumor Volume (GTV)/Clinical Target Volume (CTV). It serves as the baseline volume for dose evaluation in current clinical practice.

HI_ori_: The HI values calculated using Formulas 1, 2, and 3 are derived from the D_2%_, D_98%_, D_5%_, D_95%_, and D_50%_  metrics computed based on the V_PTVori_  volume.

V_PTVpro_: The proposed modified target volume in this study, defined as the residual volume after subtracting all target volumes with higher prescription doses from the analyzed target (see Formula 4). This volume aims to more accurately assess dose homogeneity within low-dose target volumes.

HI_pro_: The HI values calculated using Formulas 1, 2, and 3 are derived from the D_2%_, D_98%_, D_5%_ , D_95%_, and D_50%_  metrics computed based on the V_PTVpro_  volume.

Nested PTV: Geometric relationship where one PTV fully or partially contains another.

Dose overlap: In multi-target treatment planning, the spatially overlapping area of different target volumes where the dose distribution is influenced by multiple prescribed doses.

Boost-excluded volume: a delineated sub-volume within a target that is explicitly excluded from receiving the higher “boost” dose during a course of radiotherapy.

PTV-PTVboost: The volumetric region in BC radiotherapy planning obtained by performing boolean subtraction of PTVboost from PTV.

PTV1-all: The volumetric region in NPC radiotherapy planning obtained by performing boolean subtraction of PTVp, PTVn, and PTVrpn from PTV1.

## Materials and Methods

### Redefinition of the V_PTV_

The three main HI formulas commonly used in the literature, listed in chronological order, are:

(1) The HI formula proposed by Wu et al. (2003) was

(1) \begin{eqnarray*}\mathrm{HI}=({\mathrm{D}}_{2\%}-{\mathrm{D}}_{98\%})/{\mathrm{D}}_{\mathrm{p}}\times 100\%\end{eqnarray*}

where 2% and 98% represent the doses to 2% and 98% volume of the total PTV respectively, and D_*p*_ denotes the prescribed dose for the PTV. In this study, the HI value calculated using Formula 1 is denoted as HI_ori1_. Lower HI_ori1_ values are indicative of a more homogeneous target dose.

(2) The HI formula proposed by Wang et al. (2005) was

(2) \begin{eqnarray*}\mathrm{HI}={\mathrm{D}}_{5\%}/{\mathrm{D}}_{95\%}\end{eqnarray*}

where 5% and 95% represent the doses to 5% and 95% volume of the total PTV respectively. In this study, the HI value calculated using Formula 2 is denoted as HI_ori2_. Lower HI_ori2_ values are indicative of a more homogeneous target dose.

(3) The HI formula proposed by ICRU Report 83 was

(3) \begin{eqnarray*}\mathrm{HI}=({\mathrm{D}}_{2\%}-{\mathrm{D}}_{98\%})/{\mathrm{D}}_{50\%}\times 100\%\end{eqnarray*}

where 2% and 98% represent the doses to 2% and 98% volume of the total PTV respectively. 50% represent the doses to 50% volume of the total PTV. In this study, the HI value calculated using formula 3 is denoted as HI_ori3_. Lower HI_ori3_ values are indicative of a more homogeneous target dose.

**Figure 1:**
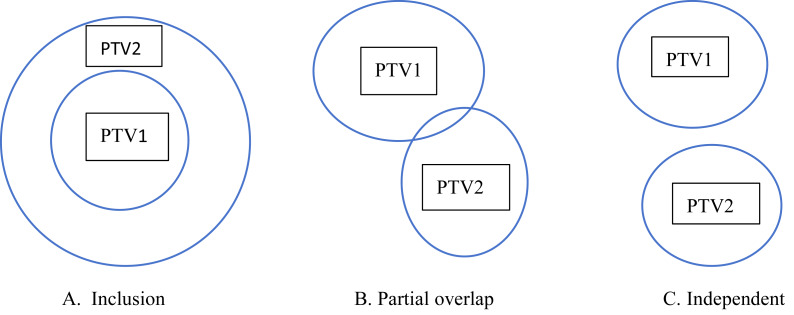
Three types of relationship between PTV1 and PTV2.

For single target plan, the D_2%_, D_98%_, D_5%_, D_95%_ and D_50%_ of PTV in the Formulas 1–3 are contributions from the PTV itself and the HI value calculated from Formulas 1–3 can truly represent the homogeneity of the target. For two targets plan such as PTV1 and PTV2, there may be three types of relationships shown in Fig. 1, which are inclusive, partially overlap, and completely independent. For the independent relationship between PTV1 and PTV2, regardless of whether the prescription doses are identical between the two target volumes, or the partial overlap relationship between PTV1 and PTV2 with the same prescription dose, all values from D_2%_ to D_98%_ used in Formulas 1–3 for either target remain unaffected by the dose distribution of the other target. Consequently, the derived HI_ori1_, HI_ori2_, and HI_ori3_ values are entirely independent of the other target’s dose characteristics, ensuring these indices accurately reflect the intrinsic dose homogeneity of the analyzed target volume. But for the partial overlap relationship between PTV1 and PTV2 with the different prescription dose, or the inclusion relationship between PTV1 and PTV2 with the different prescription dose (the PTV1 and PTV2 with inclusion relationship can only have different prescription doses), for target volume with lower prescription doses, the calculated HI values may become distorted (artificially inflated) because from the D_2%_ to D_98%_ dose parameters can be influenced by nearby high-prescription-dose target volume. However, the HI values of high-prescription-dose targets remain unaffected by the dose distribution of low-prescription targets. For plans with more than 2 target volumes such as NPC plan, the relationships between the targets and the prescription dose are more complex and the HI value is also distorted for low-dose target volumes which contains high-dose target volumes.

In view of the above analysis, we need to redefine V_PTV_ so that it can analyze the true HI value of certain target volume which contains high-dose target volumes in the multi-target plan, so that its HI value can truly reflect the homogeneity of the analyzed target, and eliminate the influence of high-dose target volumes. The new V_PTV_ of PTV can be defined as:

(4) \begin{eqnarray*}{\mathrm{V}}_{\mathrm{PTV}}=\mathrm{PTV}-\sum _{\mathrm{i=1}}^{\mathrm{k}}{\mathrm{PTV}}_{\mathrm{ i}}\end{eqnarray*}

where ${\mathop{\sum }\nolimits }_{\mathrm{i=1}}^{\mathrm{k}}{\mathrm{PTV}}_{\mathrm{i}}$ represents all the target volumes where the prescribed dose is higher than the PTV. - represents the subtraction of boolean operations. The original PTV was designated as V_PTVori_, while the PTV calculated using Formula 4 was termed V_PTVpro_.

Based on the V_PTV_ definition in this study, the HI values calculated according to Eqs. (1)–(3) are denoted as HI_pro1_, HI_pro2_, and HI_pro3_, respectively.

### Patients’ characteristics

Fifteen cases of BC with conserving surgery and fifteen NPC cases were retrospectively selected to evaluate the performance of HI_pro1_, HI_pro2_, and HI_pro3_. This retrospective study was approved by Chinese Academy of Medical Sciences Cancer Hospital Shenzhen Hospital Ethics Committee (No. JS2023-17-1 and and No. JS2024-30-1) and complied with the Declaration of Helsinki. The informed consent form template are provided in the supplementary materials. Written informed consent was obtained from all patients involved in the study. These cases of BC and NPC were previously treated with VMAT technique in our institution. All patient images were obtained using the GE Discovery RT590 CT simulator. BC cases had five mm slice spacing and NPC cases had 2.5 mm slice spacing. All BC cases had the target of PTVboost and PTV with the prescription dose of 49.5Gy, 43.5Gy in 15 fractions (Wang et al., 2019a). NPC cases had the target of PTVp, PTVn, PTVrpn, PTV1 and PTV2 with the prescription dose of 69.96Gy, 69.96Gy, 69.96Gy, 60.06Gy and 54.45Gy in 33 fractions (Wang et al., 2019b). Table 1 lists the basic and clinical characteristics of BC patients included in the study and Table 2 lists the basic and clinical characteristics of NPC patients.

**Table 1:** Basic and clinical characteristics of conserving surgery BC patients (*n* = 15).

Gender				
Male	0			
Female	15			
	Age (years)	PTVboost (cc)	PTV (cc)	PTV-PTVboost (cc)
Mean	53.1	126.5	618.3	488.6
Median	55	127.9	599.4	467.0
Range	41–62	76.3–201.8	257.0–1,257.6	175.7–1,068.7

**Notes.**

PTVboost (cc), PTV (cc), and PTV-PTVboost (cc) represent the volumes of PTVboost, PTV, and PTV-PTVboost, respectively. PTV-PTVboost represents the boolean operation result of PTV minus PTVboost in the Monaco treatment planning system.

**Table 2:** Basic and clinical characteristics of NPC patients.

Gender							
Male	13						
Female	2						
	Age (years)	PTVp (cc)	PTVn (cc)	PTVrpn (cc)	PTV1 (cc)	PTV1-all (cc)	PTV2 (cc)
Mean	52.5	61.2	37.6	14.2	537.8	429.0	122.4
Median	54	53.8	40.3	12.6	541.8	434.0	125.2
Range	31–69	36.2–104.9	8.7–67.3	3.8–60.0	316.6–786.9	256.9–686.6	18.1–232.9

**Notes.**

PTVp (cc), PTVn (cc), PTVrpn (cc), PTV1 (cc), PTV1-all(cc) and PTV2(cc) represent the volumes of PTVp, PTVn, PTVrpn, PTV1, PTV1-all and PTV2, respectively. PTV1-all represents the boolean operation result of PTV1 minus (PTVp, PTVn, and PTVrpn) in the Monaco treatment planning system.

### Treatment planning

All treatment plans were designed using the Monaco planning system. For each breast cancer patient, the plan initially employed two opposing conformal fields to deliver 80% of the prescription dose to the PTV, followed by two partial VMAT arcs to achieve the full prescription dose coverage for PTVboost and PTV. The VMAT optimization utilized Monaco’s Autoflash functionality (Wang et al., 2021; Clements et al., 2018; Rossi et al., 2021). For each NPC patient, the plan was generated using three full arcs with VMAT. The planning goal was to deliver a prescription dose to at least 95% of the target volume, while at the same time keeping the dose to the organs at risk and normal tissue as low as possible. Boolean-derived volumes were generated during the planning design phase. To control hot spots in the lower-prescription target volume (defined as doses exceeding 1.1 times its prescription), we used the lower-prescription target volume minus a margin value of the higher-prescription target volume, and the corresponding optimization objectives were also based on this subtracted-and-expanded structure.

For the BC plans in this study, since V_PTVboost_ does not contain any higher-prescription sub-volume, according Formula 4, we have V_PTVboostpro_ = V_PTVboostori_, whereas V_PTVpro_ = V_PTVori_ − V_PTVboostori_ (where − denotes the Boolean subtraction operation). Similarly, for the NPC plans in this study: V_PTVppro_ = V_PTVpori_, V_PTVnpro_ = V_PTVnori_, V_PTVrpnpro_ = V_PTVrpnori_, V_PTV1pro_ = V_PTV1ori_ − V_PTVpori_ − V_PTVnori_ − V_PTVrpnori_, (where − denotes the Boolean subtraction operation) V_PTV2pro_ = V_PTV2ori_. All Boolean operations are performed in Monaco planning system. Based on the V_PTVpro_ and V_PTVori_ volume, dose-volume calculations were carried out to obtain the D_2%_, D_5%_, D_50%_, D_95%_, D_98%_ for all targets of all the BC and NPC patient’s plans in Monaco for both V_PTVpro_ and V_PTVori_, respectively. The HI_ori1_ and HI_pro1_, HI_ori2_ and HI_pro2_, HI_ori3_ and HI_pro3_ were calculated from the collected data using the corresponding calculation Formulas 1, 2 and 3.

### Statistical analysis

#### Basic statistical analysis

All statistical analyses were performed using SPSS 25.0 (IBM Corp., Armonk, NY, USA). Continuous variables are presented as mean ± standard deviation. To compare differences between V_PTVori_ and V_PTVpro_, as well as between HI_ori_ and HI_pro_, a paired two-tailed non-parametric Wilcoxon signed-rank test was employed. A *p*-value less than 0.05 was considered statistically significant.

#### Correlation analysis and effect size calculation

To assess agreement between conventional and modified methods, Pearson correlation analysis was conducted for three pairs of HI (HI_ori1_∼HI_pro1_, HI_ori2_∼HI_pro2_, HI_ori3_∼HI_pro3_). The following were calculated:

(1). Pearson correlation coefficient (r) and its 95% confidence interval: Confidence intervals were calculated using the Fisher Z transformation method, which is appropriate for estimating correlation coefficient intervals with small sample sizes.

(2). Effect size: Cohen’s d was used to standardize mean differences, calculated as d = $ \frac{\overline{X1}-\overline{X2}}{{S}_{pooled}} $, where s_pooled_ is the pooled standard deviation. Effect sizes were interpreted according to Cohen’s conventions (*d* = 0.2 small, 0.5 medium, 0.8 large effect).

#### Reliability and agreement analysis

To provide a more comprehensive assessment of measurement agreement, the following analyses were supplemented:

(1). Intraclass Correlation Coefficient (ICC):

ICC was calculated using a two-way random effects model to evaluate measurement agreement between conventional and modified methods. 95% confidence intervals for ICC values were computed. ICC > 0.75 indicates good reliability, and >0.9 indicates excellent reliability.

(2). Bland–Altman Agreement Analysis:

Mean difference (Bias) was calculated, representing the average difference between the two measurement methods. Limits of Agreement (LoA) were computed: LoA = Bias ± 1.96 × SD_diff_, where SD_diff_ is the standard deviation of the differences. LoA width reflects measurement variability at the individual case level.

#### Comparative analysis of formula performance

To objectively compare the performance differences among the three HI formulas, the following analyses were conducted:

(1). Statistical Testing of Correlation Coefficient Differences:

Meng, Rosenthal & Rubin’s (1992) test was used to compare differences between dependent correlation coefficients based on the same sample. Bootstrap resampling (1,000 repetitions) was employed to verify result robustness and calculate 95% confidence intervals for correlation coefficient differences. Differences between Formulas 1 and 2, Formulas 1 and 3, and Formulas 2 and 3 correlation coefficients were compared separately.

#### Statistical analysis for clinical validation study

For the clinical validation study described in Section 3.6:

(1). Expert Consensus Analysis: The proportion of experts selecting V_PTVpro_ was calculated, and qualitative feedback reasons provided by experts were reported.

(2) Clinical Plan Selection Analysis: Consistency patterns in expert plan selection tasks were reported descriptively. Due to high consensus among experts, no further statistical agreement tests were performed.

#### Missing data handling and statistical assumptions

Missing values in correlation analyses were handled using pairwise exclusion to maximize utilization of available data. Normality assumptions were assessed for all statistical tests, with continuous variables evaluated using Shapiro–Wilk test. 95% confidence intervals for key statistical measures were reported to provide estimation precision information.

### Clinical validation study

To evaluate the clinical relevance and applicability of the proposed method, we conducted a comprehensive validation study comprising two integrated components: (1) an expert consensus survey on conceptual rationality, and (2) a practical clinical plan selection task. This dual approach allowed us to assess both the theoretical validity and the potential practical impact of the modified HI in a simulated decision-making context.

#### Expert panel formation

An independent panel of six senior radiotherapy professionals was convened for this validation. All panelists were from our institution, had no prior involvement in or knowledge of this study, and declared no conflicts of interest. The panel was structured to include balanced expertise:

Radiotherapy Oncologists: three attending physicians or consultants (associate senior title or above), each with over 10 years of clinical experience in treatment planning and evaluation.

Medical Physicists: three senior medical physicists (associate senior title or above), each with over 10 years of hands-on experience in treatment planning, optimization, and quality assurance.

#### Conceptual rationality assessment

A structured questionnaire was administered to the expert panel to assess the conceptual rationale underlying the proposed method. The central question was designed to elicit their professional judgment on the fundamental approach: When evaluating the dose homogeneity specifically within a lower-dose target volume (*e.g.*, BC PTV or NPC PTV1) that geometrically encompasses a higher-dose boost volume, which target volume definition is more appropriate for calculating the HI. Experts were asked to choose between:

V_PTVori_: The complete, original target volume.

V_PTVpro_: The volume remaining after the boolean subtraction of all higher-prescription-dose targets (as defined in this study).

The survey was conducted anonymously to ensure unbiased responses.

#### Simulated clinical plan selection task

To investigate whether the difference between HI_ori_ and HI_pro_ could influence clinical preference in a controlled scenario, we designed a plan comparison exercise:

(1). Case Selection: Three representative BC cases and three NPC cases were randomly selected from the study cohort.

(2). Generation of Comparator Plans: For each selected case, a new treatment plan (plan2) was created alongside the original clinically accepted plan (plan1). The generation of plan2 followed a specific protocol:

Primary Objective: Introduce a discernible difference in the homogeneity of the lower-dose target volume (PTV for BC, PTV1 for NPC) compared to plan1. This was achieved by deliberately relaxing the homogeneity optimization constraints for these specific targets during the planning of plan2.

Secondary Constraint: Every effort was made during the re-optimization to minimize concomitant changes to other critical plan metrics. The goals were to maintain comparable doses to organs at risk (OARs), similar target volume coverage (D_95%_ ), and comparable conformity indices between plan1 and plan2. We acknowledge the intrinsic challenge in completely isolating one planning objective, as adjustments inevitably affect the overall dose distribution.

(3). Blinded Expert Evaluation: The two plans (plan1 and plan2) for each of the six cases were presented to the expert panel in a blinded and randomized order. Experts were instructed to review all standard clinical evaluation tools (dose-volume histograms, dose distributions on computed tomography slices) and, based solely on their clinical experience and judgment, select the plan they considered more suitable for treatment for each case. They were not informed which plan was the original or which had modified homogeneity.

(4). Data Collection: each expert’s choice for every case was recorded. Subsequently, the plans were ranked for each case based on both the HI_ori_ and HI_pro_ values (lower HI indicating better ranking). These rankings were then compared to the expert-selected “preferred” plan to assess alignment.

#### Objectives and analysis

This validation framework aimed to:

(1). Establish whether the conceptual basis for using V_PTVpro_ (subtracting high-dose volumes) is favored by clinical experts over the traditional approach.

(2). Determine, in a scenario where HI is a distinguishing factor between otherwise similar plans, whether the plan ranking dictated by HI_pro_ correlates better with expert clinical choice than the ranking dictated by HI_ori_.

## Results

### The evaluation of V_PTVori_ and V_PTVpro_ with the BC and NPC plan

**Figure 2:**
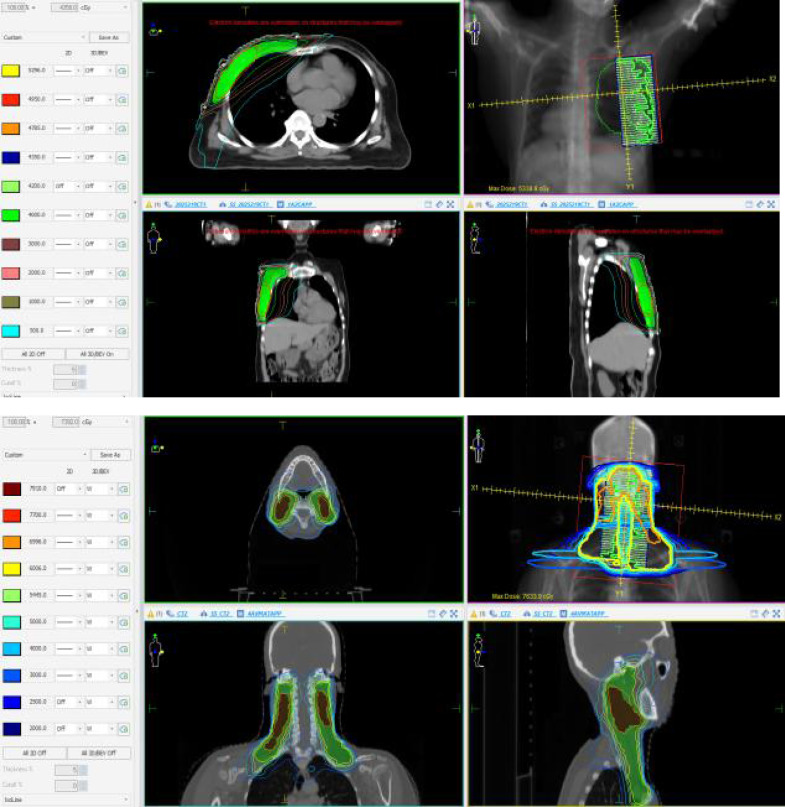
The dose distribution diagram for one BC and one NPC treatment plan.

Figure 2 shows the dose distribution of one BC and one NPC plan selected in this study. Table 3 shows the paired two-tailed non-parametric Wilcoxon signed-rank test results between V_PTVori_ and V_PTVpro_. It can be seen from the results that for BC, V_PTVori_ and V_PTVpro_ for PTVboost were identical, whereas statistically significant differences were observed between V_PTVori_ and V_PTVpro_ for PTV. Notably, the V_PTVpro_ values calculated using our proposed method were significantly smaller than the original V_PTVori_ values. For NPC cases, the V_PTVpro_ and V_PTVori_ values were identical for PTVp, PTVn, PTVrpn, and PTV2. However, statistically significant differences (*p* < 0.05) were observed between V_PTVpro_ and V_PTVori_ for PTV1, with V_PTVpro_ being significantly smaller than V_PTVori_.

**Table 3:** The comparison of V_PTVori_ and V_PTVpro_ with all PTVs.

	BC				NPC									
	PTVboost		PTV		PTVp		PTVn		PTVrpn		PTV1		PTV2	
	ori	pro	ori	pro	ori	pro	ori	pro	ori	pro	ori	pro	ori	pro
Value	126.5 ± 36.2	126.5 ± 36.2	618.3 ± 283.3	488.6 ± 252.6	61.2 ± 21.9	61.2 ± 21.9	37.6 ± 19.0	37.6 ± 19.0	14.2 ± 13.6	14.2 ± 13.6	537.8 ± 129.6	429.0 ± 122.4	122.4 ± 64.0	122.4 ± 64.0
*Z*	0.000		−3.408		−0.000		0.000		0.000		−3.408		−0.000	
*p*	1.000		0.001		1.000		1.000		1.000		0.001		1.000	

**Notes.**

ori, V_PTVori_; pro, V_PTVpro_.

### The evaluation of HI_ori1_ and HI_pro1_, HI_ori2_ and HI_pro2_, HI_ori3_ and HI_pro3_ with the BC and NPC plan

Table 4 shows the paired two-tailed non-parametric Wilcoxon signed-rank test results between HI_ori1_ and HI_pro1_, HI_ori2_ and HI_pro2_, HI_ori3_ and HI_pro3_. The results indicate that for BC: the HI_ori1_ and HI_pro1_, HI_ori2_ and HI_pro2_, HI_ori3_ and HI_pro3_ values calculated by the three formulas for the PTVboost were the same respectively. For the PTV, the HI values calculated from the three formulas, as well as the HI_ori_ and HI_pro_ values, showed statistically significant differences, with HI_pro_ being significantly lower than HI_ori_. The results indicate that for NPC, for the PTVp, PTVn, PTVrpn, and PTV2 target, the HI_ori1_ and HI_pro1_, HI_ori2_ and HI_pro2_, HI_ori3_ and HI_pro3_ values calculated by the three formulas were the same respectively. However, for the PTV1, the HI_ori_ and HI_pro_ values calculated from the three formulas previously proposed, showed statistically significant differences, with HI_pro_ being significantly lower than HI_ori_.

**Table 4:** The comparison results of HI_ori1_ and HI_pro1_, HI_ori2_ and HI_pro2_, HI_ori3_ and HI_pro3_.

		BC				NPC									
		PTVboost		PTV		PTVp		PTVn		PTVrpn		PTV1		PTV2	
		HI_ori_	HI_pro_	HI_ori_	HI_pro_	HI_ori_	HI_pro_	HI_ori_	HI_pro_	HI_ori_	HI_pro_	HI_ori_	HI_pro_	HI_ori_	HI_pro_
1 (%)	Value	7.294 ± 0.959	7.294 ± 0.959	21.55 ± 1.212	16.28 ± 1.761	7.755 ± 1.473	7.755 ± 1.473	8.714 ± 1.558	8.714 ± 1.558	8.467 ± 1.883	8.467 ± 1.883	25.65 ± 1.81	22.62 ± 1.81	18.95 ± 2.01	18.95 ± 2.01
	*Z*	0.000		−3.408		0.000		0.000		0.000		−3.408		0.000	
	*P*	1.000		0.001		1.000		1.000		1.000		0.001		1.000	
2	Value	1.056 ± 0.006	1.056 ± 0.006	1.188 ± 0.010	1.119 ± 0.017	1.058 ± 0.010	1.058 ± 0.010	1.068 ± 0.013	1.068 ± 0.013	1.065 ± 0.012	1.065 ± 0.012	1.211 ± 0.014	1.176 ± 0.016	1.137 ± 0.018	1.137 ± 0.018
	*Z*	0.000		−3.408		−0.000		0.000		0.000		−3.408		−0.000	
	*P*	1.000		0.001		1.000		1.000		1.000		0.001		1.000	
3 (%)	Value	7.008 ± 0.896	7.008 ± 0.896	20.51 ± 1.09	15.60 ± 1.65	7.437 ± 1.394	7.437 ± 1.394	8.368 ± 1.474	8.368 ± 1.474	8.096 ± 1.765	8.096 ± 1.765	23.12 ± 1.57	20.68 ± 1.69	17.94 ± 1.93	17.94 ± 1.93
	*Z*	0.000		−3.408		−0.000		0.000		0.000		−3.408		−0.000	
	*P*	1.000		0.001		1.000		1.000		1.000		0.001		1.000	

**Notes.**

1(%) represents the HI value calculated using Formula 1 with units in percentage, 2 represents the HI value calculated using Formula 2, 3(%) represents the HI value calculated using Formula 2 with units in percentage. BC, Breast Cancer; NPC, nasopharyngeal carcinoma.

### The correlation of HI_ori1_ and HI_pro1_, HI_ori2_ and HI_pro2_, HI_ori3_ and HI_pro3_

**Figure 3:**
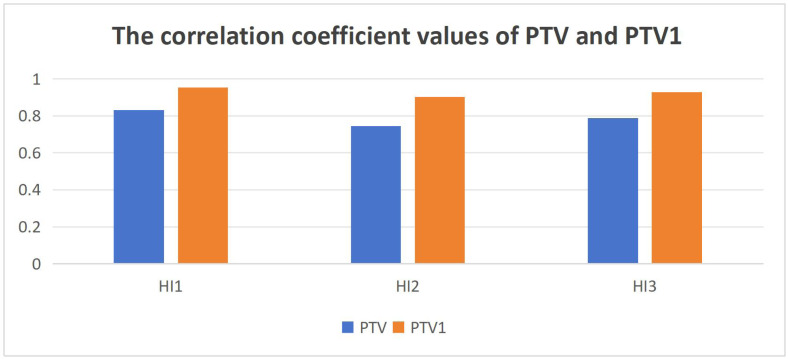
The correlation coefficient values with PTV of BC and PTV1 of NPC. HI1: the correlation coefficient of HI_ori1_ and HI_pro1_, HI2: the correlation coefficient of HI_ori2_ and HI_pro2_, HI3: the correlation coefficient of HI_ori3_ and HI_pro3_, PTV: the PTV of BC, PTV1: the PTV1 of NPC.

Table 5 shows the correlation analysis results of HI_ori1_ and HI_pro1_, HI_ori2_ and HI_pro2_, HI_ori3_ and HI_pro3_. For the PTVboost of BC and PTVp, PTVn, PTVrpn, and PTV2 of NPC, since the values of HI_ori1_ and HI_pro1_, HI_ori2_ and HI_pro2_, and HI_ori3_ and HI_pro3_ were identical for each respective pair across these five target volumes, their correlation coefficients all reached 1.000 with *p*-values of 0.000. For PTV of BC and PTV1 of NPC, the correlation coefficient values between HI_ori1_-HI_pro1_, HI_ori2_-HI_pro2_, and HI_ori3_-HI_pro3_ showed variations, though all demonstrated positive correlations with statistically significant *p*-values < 0.01. The computational results for these two target volumes revealed a consistent pattern: the HI_ori1_-HI_pro1_ pair exhibited the highest correlation coefficient values, while HI_ori2_-HI_pro2_ consistently showed the lowest values among the three method comparisons. Figure 3 presents the comparative bar charts illustrating the correlation coefficients between HI_ori1_ and HI_pro1_, HI_ori2_ and HI_pro2_, HI_ori3_ and HI_pro3_ for both PTV of BC and PTV1 of NPC. To provide more robust statistical evidence, we calculated multidimensional agreement metrics between HI_ori_ and HI_pro_ for both BC PTV and NPC PTV1 which have the different HI value between HI_ori_ and HI_pro_. These metrics included: the Pearson correlation coefficient (r) with its 95% confidence interval (CI), Cohen’s d effect size, the intraclass correlation coefficient (ICC) with its 95% CI, and the Bland–Altman bias along with the upper and lower limits of agreement (LoA). The complete statistical results for the three formulas under both clinical scenarios are summarized in Table 6. In addition, the results of direct pairwise comparisons of the correlation coefficients among the three formulas are presented in Table 7. The results showed that significant differences were found only in the comparison between Formulas (1) and (2): for BC PTV (*p* = 0.024) and NPC PTV1 (*p* = 0.031), while the remaining pairwise comparisons did not reach statistical significance (*p* > 0.2). This indicates that the difference in correlation between Formulas (1) and (2) is statistically significant in both target regions.

**Table 5:** The correlation analysis results of HI_ori1_ and HI_pro1_, HI_ori2_ and HI_pro2_, HI_ori3_ and HI_pro3_.

		BC		NPC				
		PTVboost	PTV	PTVp	PTVn	PTVrpn	PTV1	PTV2
HI1	correlation coefficient	1.000	0.831	1.000	1.000	1.000	0.953	1.000
	*P*	0.000	0.000	0.000	0.000	0.000	0.000	0.000
HI2	correlation coefficient	1.000	0.746	1.000	1.000	1.000	0.903	1.000
	*P*	0.000	0.001	0.000	0.000	0.000	0.000	0.000
HI3	correlation coefficient	1.000	0.788	1.000	1.000	1.000	0.927	1.000
	*P*	0.000	0.000	0.000	0.000	0.000	0.000	0.000

**Notes.**

HI1, the correlation coefficient of HI_ori1_ and HI_pro1_; HI2, the correlation coefficient of HI_ori2_ and HI_pro2_; HI3, the correlation coefficient of HI_ori3_ and HI_pro3_.

**Table 6:** Multidimensional agreement metrics between HI_ori_ and HI_pro_ for BC PTV and NPC PTV1.

	PTV of BC			PTV1 of NPC		
	HI1	HI2	HI3	HI1	HI2	HI3
Pearson r	0.831	0.746	0.788	0.953	0.903	0.927
r 95% CI	[0.55, 0.94]	[0.38, 0.91]	[0.47, 0.93]	[0.85, 0.98]	[0.73, 0.97]	[0.79, 0.97]
Cohen’s d	3.67	5.23	3.4	6.4	8.75	5.2
ICC	0.92	0.94	0.91	0.976	0.949	0.963
ICC 95% CI	[0.85, 0.96]	[0.88, 0.97]	[0.84, 0.95]	[0.94, 0.99]	[0.86, 0.98]	[0.90, 0.99]
Bias	0.055	0.069	0.051	0.032	0.035	0.026
SD_diff	0.01	0.009	0.01	0.005	0.004	0.005
Lower of LoA	0.035	0.05	0.031	0.022	0.027	0.016
Upper of LoA	0.075	0.086	0.071	0.042	0.043	0.038

**Notes.**

HI1, the correlation coefficient of HI_ori1_ and HI_pro1_; HI2, the correlation coefficient of HI_ori2_ and HI_pro2_; HI3, the correlation coefficient of HI_ori3_ and HI_pro3_; Pearson r, Pearson correlation coefficient; r 95% CI, 95% confidence interval of Pearson correlation coefficient; Cohen’s d, Cohen’s d effect size; ICC, intraclass correlation coefficient; ICC 95% CI, 95% confidence interval of intraclass correlation coefficient; Bias, the Bland–Altman bias; Lower of LoA, lower limits of agreement about the Bland–Altman bias; Upper of LoA, Upper limits of agreement about the Bland–Altman bias.

**Table 7:** The results of direct pairwise comparisons of the correlation coefficients among the three formulas for BC PTV and NPC PTV1.

	PTV of BC			PTV1 of NPC		
	Difference	95% CI	*P* (two-tailed)	Difference	95% CI	*P* (two-tailed)
F1 *vs* F2	0.085	[0.015, 0.155]	0.024	0.05	[0.005, 0.095]	0.031
F1 *vs* F3	0.043	[−0.022, 0.108]	0.406	0.026	[−0.015, 0.067]	0.204
F2 *vs* F3	−0.042	[−0.124, 0.040]	0.417	−0.024	[−0.082, 0.034]	0.398

**Notes.**

F1 *vs* F2, Formula 1 *vs* Formula 2; F1 *vs* F3, Formula 1 *vs* Formula 3; F2 *vs* F3, Formula 2 *vs* Formula 3; 95% CI, 95% confidence interval.

### Results of the clinical validation study

#### Expert preference regarding the evaluation volume: V_PTVpro_*vs* V_PTVori_

In the questionnaire survey investigating the preferred target volume for homogeneity assessment in multi-target plans, all six experts (100%) unanimously selected V_PTVpro_ as the more conceptually reasonable choice. This consistent preference was observed across both clinical scenarios included in this study (BC and NPC) and was shared equally by the two professional subgroups (radiotherapy oncologists and medical physicists).

Analysis of Expert Rationale and Context: Open-ended feedback revealed that this unanimous agreement aligns closely with established clinical workflows. Radiation oncologists noted that in clinical practice, when evaluating the homogeneity of a low-dose target volume encompassing a high-dose boost region, they typically disregard the already independently assessed high-dose area and focus instead on the dose distribution within the remaining volume. Medical physicists explained from a planning optimization perspective that uniformity constraints are typically applied to the residual volume (*e.g.*, “PTV minus PTVboost”) rather than to the entire original target volume. Therefore, the V_PTVpro_ calculation method proposed in this study essentially formalizes and quantifies the implicit assessment logic routinely employed by clinical experts.

#### Impact of HI differences on clinical plan selection

In the clinical plan selection validation involving six test cases, all six experts, following blinded review, unanimously selected the plan with superior homogeneity (plan1). This choice was based on a comprehensive evaluation of the overall plan quality, including target coverage, organ-at-risk sparing, and dose distribution.

However, further analysis revealed that despite the clear expert preference for plan1, the ranking outcomes based on HI_pro_ and HI_ori_ were completely concordant in this validation exercise. For every single case, the HI_pro_ value for plan1 was lower than that for plan2, and simultaneously, the HI_ori_ value for plan1 was also lower than that for plan2. In other words, within the specific plan set generated for this validation, the two calculation methods exhibited identical trends in ranking the plans from better to worse homogeneity.

## Discussion

The HI provides an effective tool for analyzing dose homogeneity within target volumes, serving as a valuable reference for comparing different treatment plans for the same patient in clinical practice. At present, the HI value has become one of the basic parameters for dosimetric comparison literature and is widely used in the evaluation of clinical treatment plans. Meanwhile, the HI values of each target volume may guide us on how to modify the plan. Therefore, it is crucial for the HI value to accurately reflect the homogeneity of the PTV. At the same time, the number of target volumes varies for each tumor disease, which may be single or multiple targets. The relationship between each target in multi-target tumors is complex, and the prescription dose for each target may also be different. In current dosimetric comparison studies, the widely used HI calculation formulas are the three mentioned previously (Formulas 1 to 3). When analyzing the 2%, 5%, 50%, 95%, and 98% dose values of the target volume, these formulas all use the entire target volume V_PTV_. For single-target plans or multi-target plans with independent relationships (Fig. 1C), or multi-target plans with inclusive/partially inclusive relationships where the prescription dose is high (Figs. 1A & 1B), the V_PTV_ in Formulas 1–3 represents the true target volume, ensuring that the derived 2%, 5%, 50%, 95%, and 98% values are accurate. Consequently, the HI values calculated using Formulas 1–3 are physically realistic. However, when applied to low-dose targets within inclusive/partially inclusive relationships (Figs. 1A & 1B), the V_PTV_ in these formulas erroneously includes the volume of high-dose targets, leading to distortions in the 2%, 5%, 50%, 95%, and 98% dose values of the low-dose targets. As a result, the calculated HI values become artificially skewed. The guidance for dosimetric comparison or clinical plan modification based on this HI value is incorrect. Therefore, this article revised the formula for V_PTV_ which shown in Formula 4, so that when dealing with different numbers of target areas and different prescription doses in clinical planning, its V_PTV_ should only include the volume range of the analysis target, and remove the influence of other target areas on the analysis target area. The calculated HI value can accurately reflect the homogeneity index of the analyzed target area.

From the V_PTVpro_ calculation formula proposed in this study, it can be seen that for single target volume, such as conventional rectal cancer, cervical cancer without lymph node involvement, lung cancer, esophageal cancer and liver cancer, or multi-target volume plans with completely independent or partial overlap relationships with the same prescription dose such as PTVp, PTVn, PTVrpn, PTV2 of NPC, the V_PTVpro_ proposed in this study is completely consistent with the original V_PTVori_. Consequently, the D_2%_, D_5%_, D_50%_, D_95%_, D_98%_ for V_PTVpro_ and V_PTVori_ are also identical, leading to entirely consistent HI_pro_ and HI_ori_ results calculated by the three formulas. Therefore, the method proposed in this article is also applicable to single-target treatment plans. This equivalence is clearly demonstrated by the computational results for the PTVboost of BC and PTVp, PTVn, PTVrpn, and PTV2 of NPC in the study. For the two target areas or multi-target volume plans with inclusion relationship such as PTV of BC and PTV1 of NPC patients selected in this article, there exists a statistically significant difference between V_PTVpro_ and V_PTVori_, with V_PTVpro_ being consistently smaller than V_PTVori_. This discrepancy occurs because V_PTVpro_ excludes the high-dose target volume within the PTV, whereas V_PTVori_ retains the entire PTV volume. The HI_pro1_, HI_pro2_ and HI_pro3_ values calculated using the proposed V_PTVpro_ method demonstrate statistically significant differences compared to their conventional counterparts HI_ori1_, HI_ori2_ and HI_ori3_. H1_pro1_ was significantly lower than HI_ori1_, HI_pro2_ was significantly lower than HI_ori2_, and HI_pro3_ was significantly lower than HI_ori3_. This result is quite understandable because for low-dose target volumes containing high-dose target volumes, after subtracting the high-dose target volume, the target homogeneity should be improved compared to the results without subtracting the high-dose target volume. As demonstrated in this study, for both the PTV of BC and the PTV1 of NPC, the HI_pro_ values calculated by all three formulas were lower than the HI_ori_ values, respectively. For two or multiple target volume plans with a partial overlap relationship, if the prescription doses differ, the V_PTVpro_ for the low-prescription-dose target volume is significantly smaller than V_PTVori_. This discrepancy occurs for the same reason: V_PTVpro_ excludes the high-dose target volume within the PTV, whereas V_PTVori_ retains the entire PTV volume. Consequently, the HI_pro1_, HI_pro2_, and HI_pro3_ values calculated using the proposed V_PTVpro_ method are also significantly lower than the corresponding HI_ori1_, HI_ori2_, and HI_ori3_ values.

This study conducted a correlation analysis on the HI_ori_ and HI_pro_ of all target volumes of the selected plans, as shown in Tables 5, 6 and 7 and Fig. 3. Here are the results showing that all three formulas exhibit a positive correlation between HI_ori_ and HI_pro_, with p-values all less than 0.01. For PTV of BC and PTV1 of NPC, the correlation coefficients vary slightly among the three formulas. HI_ori1_ and HI_pro1_ of formula 1 shows the highest correlation coefficient, exceeding 0.8, indicating a very strong correlation. HI_ori2_ and HI_pro2_ of formulas 2, HI_ori3_ and HI_pro3_ of formulas 3 demonstrate strong correlations (0.6−0.8) for PTV of breast cancer, but a very strong correlation (>0.8) for PTV1 of nasopharyngeal cancer. From these findings, we can conclude that Formula (1) is the most agreement in analyzing HI variations for multi-target volumes with low-dose targets minus high-dose volumes in this study. Although all three formulas correctly reflect the trend of HI changes in the study, Formula (1) exhibits the strongest agreement. Therefore, among the three widely used formulas for calculating HI, we recommend that Formula 1) may be more appropriate for clinical practice when evaluating target homogeneity. For patients with multiple targets and prescription doses, when comparing HI values among multiple candidate treatment plans, the results obtained using the original calculation method of these three formulas demonstrate stronger correlation and better consistency with the results derived from the proposed method in this study.

We believe it may be more reasonable and accurate to calculate the HI for the remaining volume of the low-prescription-dose target after subtracting the high-prescription-dose target volume within it. First, for high-prescription-dose target volumes, the HI values have already been analyzed separately for their own volumes. If the low-dose target volumes—which partially or fully include these high-dose regions—are analyzed without excluding the high-dose target volume, this would lead to unreasonable double-counting of the high-dose regions. Second, in clinical treatment planning, when setting dose constraints for low-dose targets, it is standard practice to subtract the high-dose volumes before applying optimization objectives. Finally, by analyzing the HI values of low-dose targets after subtracting the high-dose regions, we can more accurately and intuitively assess the dose homogeneity of the low-dose volumes of interest, which is the key focus for physicist and radiation oncologist. Therefore, the original methods for calculating HI values may overestimate the HI in target volumes with lower prescription doses within multi-target plans.

The clinical validation results of this study provide further practical support for this concept. First, at the level of assessment philosophy, all participating experts unanimously agreed that the method based on V_PTVpro_ is more reasonable, reflecting the underlying logic commonly followed in clinical practice when evaluating HI in multi-target plans, namely, avoiding double assessment of high-dose regions. Second, at the level of practical decision-making, since the ranking trends of HI_pro_ and HI_ori_ for plan quality were completely consistent in this validation, the new method did not alter the final plan selection. This finding further clarifies that the primary value of this study lies not in overturning clinical decision-making workflows, but in providing a more accurate and reasonable quantitative descriptive tool. This tool can identify and correct the potential systematic overestimation bias that may exist when using conventional methods to calculate the homogeneity of target volumes with lower prescription doses. Therefore, we recommend adopting the homogeneity assessment method based on V_PTVpro_ in contexts requiring precise and comparable quantitative reporting, such as multicenter clinical trials, comparative studies of radiotherapy techniques, or departmental quality control.

The V_PTVpro_ calculation method proposed in this study aligns closely with the intrinsic need for dose-level-specific evaluation in current clinical practice for multi-target radiotherapy. The International Commission on Radiation Units and Measurements (ICRU) explicitly stipulates in its Report No. 83 that for plans employing simultaneous integrated boost (SIB) techniques, dosimetric reporting must be performed independently for the target volume corresponding to each prescription dose level (International Commission on Radiation Units and Measurements (ICRU), 2010). Similarly, in post-breast-conserving surgery radiotherapy, authoritative consensus guidelines also distinctly separate the whole breast target volume and the tumor bed boost target volume as two independent target structures (Offersen et al., 2015). More importantly, in the latest research on simultaneous integrated boost planning for partial breast irradiation, the structure defined as “PTV_Eval minus Boost_Eval” (PTV-BoostEval) was explicitly adopted to delineate the evaluation volume for the low-dose target, with key dose parameters (such as D_2%_) constrained and optimized based on this structure (Burton et al., 2024). These standards and practices collectively establish a clear principle: when evaluating the dose distribution of a low-dose target volume, the high-dose subvolume contained within it may be excluded to obtain results that truly reflect the distribution at its own prescription dose level. The V_PTVpro_ proposed in this study precisely formalizes, generalizes, and systematically integrates this widely recognized principle into the homogeneity index calculation framework as a quantitative tool.

For low-dose target volumes containing high-dose target subregions, there may be significant differences in prescription doses between the two targets, and the ratio of prescription doses may vary across different disease types. In this study, we deliberately did not apply margin expansion to the high-dose target volume when subtracting it from the low-dose target volume. This approach was adopted because we aimed to ensure that all target volume components are included in the HI analysis. If we were to expand the high-dose target volume during subtraction, the expanded margin region would not belong to either target volume and would consequently be excluded from analysis. Although this methodology may result in the HI values of low-dose targets being influenced by the adjacent high-dose targets, it preserves the integrity of complete dose coverage assessment across all designated target volumes.

It is important to note that HI to the target volume is only one aspect of clinical evaluation. Other characteristics, such as conformity index (CI) (Van’t Riet et al., 1997; Paddick, 2000) and dose limitation of OARs, also needed to be evaluated by the physicist. Meanwhile, the HI and other parameters such as CI can measure the quality of radiotherapy in most cases, but target volume coverage, visual inspection of CT layer by layer, and dose volume histogram are still essential.

This study has several limitations. First, the investigation of the respective correlations between HI_pro_ and HI_ori_ for the three formulas is based on the assumption that the dose distribution within the high-dose target volume does not affect the HI assessment of the low-dose target volume. Second, the potential influence of margins around the high-dose target and dose gradients was not considered. Finally, this study is based on photon plans, and its applicability to proton or heavy-ion plans may require further validation.

## Conclusion

In this study, we introduced a new V_PTV_ calculation formula from HI to calculate the homogeneity of the analyzed target volume in the case of multiple target areas. The new V_PTV_ subtracts the high-dose target volume within the low-dose target volume, aiming to provide a more reasonable and accurate reflection of the HI value for the analyzed low-dose target volume than previous formulas. Additionally, the new V_PTVpro_ proposed in this paper is also applicable for HI calculation in single-target volume. We recommend using the V_PTVpro_ proposed in this study to calculate the HI value of low-dose target volumes that partially or fully contain high-dose targets in multi-target plans, such as the PTV in breast-conserving plans and PTV1 in NPC cases selected in this study. Meanwhile, we recommend employing formula 1 for the calculation of the target HI among the three formulas.

## Supplemental Information

10.7717/peerj.21005/supp-1 Supplemental Information 1 The original data The following parameters were collected and compared for all target volumes from 15 breast cancer patients and 15 nasopharyngeal carcinoma patients in the data: Volume of PTVori and PTVpro Dose metrics: D2%, D5%, D50%, D95%, D98% Calculated HI: HI1ori and HI1pro, HI2ori and HI2pro, HI3ori and HI3pro

## Additional Information and Declarations

### Data Availability

The following information was supplied regarding data availability: The raw measurements are available in the Supplemental File.
